# Retrospective Analysis of Rechallenge with Ipilimumab in Patients with Metastatic Melanoma

**DOI:** 10.1155/2021/5531864

**Published:** 2021-07-05

**Authors:** A. A. Formozo, J. R. Gomes, R. A. Schmerling, A. C. Buzaid

**Affiliations:** Clinical Oncologist, A Beneficência Portuguesa de São Paulo, São Paulo, Brazil

## Abstract

**Background:**

Checkpoint inhibitors are effective in the treatment of several types of cancer, either being used separately or in combination. Ipilimumab pioneered the treatment of metastatic melanoma, and nowadays, it has been used more frequently in combination with anti-PD-1. Since the development of anti-PD1 for melanoma, rechallenge with ipilimumab has not been considered, although its use was considered in early trials.

**Cases:**

In this study, we analyzed 22 patients with metastatic melanoma who had benefited from the first treatment with ipilimumab, but eventually had progressive disease. They received ipilimumab at the same dose as the first treatment. Most of the patients received the second course after six months or more from the first treatment with ipilimumab. The median progression-free survival (mPFS) of the treatment with ipilimumab was 8.9 months, and the median progression-free survival of the second course was 6.3 months.

**Conclusion:**

There are limited data on rechallenge with ipilimumab addressing progression-free survival (PFS). In our analysis, twenty-two patients treated with a second course of ipilimumab were analyzed and most of them had a significant benefit. Despite the current alternatives for salvage therapies, rechallenging with ipilimumab might be an alternative to be considered in patients who had initial benefit.

## 1. Introduction

Checkpoint inhibitors have a growing importance in many types of cancer. Ipilimumab, an anti-CTLA-4 (*cytotoxic T-lymphocyte-associated-protein 4*) was the first drug in this class. Ipilimumab prevents the CTLA-4 protein (present in cytotoxic T lymphocytes) from binding to the B7 molecule (present in the antigen-presenting cells), reducing the negative signal on lymphocyte activation and, thus, increasing immune response and leading to antitumor activity [1]. Although its efficacy has been overcome by anti-PD-1 agents (*programmed cell death-1*), nearly 20% of patients had long-term overall survival in the ipilimumab's pivotal trial and in following trials and series [2–8]. Until then, ipilimumab was considered the standard first-line therapy for metastatic melanoma.

Most of the patients who had clinical benefit with ipilimumab experienced progressive disease after some time. Thus, there was a severe disease but very limited treatment options, and then, rechallenge was the best one for them [9–11]. The main purpose of this report is to bring up the importance of rechallenge on anti-PD1 combination.

## 2. Method

This is a retrospective study which evaluated patients with metastatic melanoma who had been treated in a single institution in Brazil, from April 2007 to March 2015. We analyzed patients who received ipilimumab (3 mg/kg) and had clinical benefit, characterized by objective response or stable disease with at least 6 months of duration, followed by progressive disease and treated again with ipilimumab. Patients with prior history of grade 3 or 4 toxicity were not eligible for rechallenge. The decision of a new course with ipilimumab instead of another alternative (when these became available) was made according to physicians and patients' preference, defined on individual basis.

Patients were classified according to BRAF mutation status (positive, negative, or unknown) and melanoma type (cutaneous, uveal, acral, mucosal, or primary unknown).

Response was assessed with computed tomography (CT) or positron emission tomography (PET-CT). The response was determined according to the criteria of the RECIST (Response Evaluation Criteria in Solid Tumors) version 1.1, being classified in CR, PR, SD, or PD. Progression-free survival (PFS) and overall survival (OS) were established from the first day of the second course of ipilimumab.

## 3. Case Series

We evaluated 199 patients with metastatic melanoma treated with ipilimumab from April 2007 to March 2015 (data analyzed in October 2020). Among these, twenty two (11%) received ipilimumab at the time of progression, after they had had prior benefit with the same treatment. Ten patients were male, and the median age was 59 years (range 39–85). Ten patients had cutaneous melanoma, six acral, two mucosal, and two had ocular melanoma. Other two patients had unknown primary site (Table 1). Five patients had BRAF mutation, and five had no information on this mutation, including choroidal melanomas (Table 2). The median progression-free survival (mPFS) for the first course of ipilimumab was 9.4 months (3.5 to 28.7). Most patients (86%) received the second course after an interval of six months or more (Table 3).

Before the first course of ipilimumab, most patients (86%) had received at least one treatment (Table 4). Twenty patients (91 percent) had already received another type of treatment prior to rechallenge. Only one patient received BRAF inhibitor between the courses of ipilimumab. No patient received anti-PD-1 before neither the first nor the second course of ipilimumab.

The median PFS for rechallenge was 8.8 months (range 1.3 to 75.1). No unexpected toxicity was observed. At the time of the analysis (October 2020), eight patients (36%) who received a second course of ipilimumab were alive (five of them did not receive any other treatment following after ipilimumab). Among the patients who had benefit with the second course of ipilimumab, five were treated again with ipilimumab at the time of new PD (between two and four courses of treatment in addition to the first rechallenge).

Thirty-six percent of the patients had PD at the rechallenge. Objective response rate was 27%, and clinical benefit was observed in 63% (SD in 36%, PR in 18%, and CR in 9%). Among the two patients (9%) who had CR with rechallenge, one also had it at the first course of ipilimumab. Overall survival after rechallenging with ipilimumab ranged from 11.6 to 135.5 months. Five patients were alive at the time of analysis (three of them did not receive any other treatment for melanoma than ipilimumab rechallenge, one received pembrolizumab after ipilimumab failure, and the other failed checkpoint inhibitor and is receiving BRAF inhibitor). Four patients died due to melanoma progression, and one patient was lost to follow-up in 2017 (Table 5).

## 4. Conclusions

Although new courses of ipilimumab after progressive disease among patients who benefited were considered in the pivotal trial, there are limited data on this strategy. Three studies addressed this issue, although they reported the response rate (from 12 to 23%) but not progression-free survival, nor overall survival, after rechallenge.

Despite being a retrospective study and its limitations (mainly potential selection and information biases), there were relevant results in our analysis. Twenty-two patients had benefited from the treatment of melanoma with ipilimumab, and they received a new course of treatment upon progressive disease. Most of the patients had a long PFS after this (with five patients still alive at the time of analysis). Notably, the benefit of the new course was observed only in those who had a PFS greater the six months with the first course.

## 5. Discussion

Anti-PD1 overcame ipilimumab activity with less toxicity and became the preferential alternative for first-line therapy among the patients who considered a single-agent immunotherapy. It is expected also that a significant proportion of patients who received BRAF/MEK inhibitors might need immunotherapy. Although the data for salvage of anti-PD1 failures with the combination of ipilimumab and anti-PD1 are not fully established, we believe that this might become the future standard. Still, there might be patients who will not be eligible for the combination and ipilimumab could be an alternative to be considered. It also might be an alternative for patients who progress after the combination of BRAF/MEK/anti-PD1. For this select group of patients, who may benefit from ipilimumab alone, rechallenge could still be an alternative upon progression.

## Figures and Tables

**Table 1:** Primary site.

Primary site of melanoma	Number of cases (%)
Cutaneous	10 (45)
Acral	6 (27)
Choroidal	2 (9)
Mucosal	2 (9)
Unknown	2 (9)

**Table 2:** BRAF mutation status.

BRAF mutation	Number of cases (%)
Present	6 (27)
Absent	12 (54)
Unknown	4 (18)

**Table 3:** PFS between challenge and rechallenge.

Time to progression after first ipilimumab treatment	Number of cases (%)
<6 months	3 (14)
6–12 months	15 (68)
>12 months	4 (18)

**Table 4:** Treatments before ipilimumab.

Treatment before first ipilimumab treatment	Number of cases (%)
Chemotherapy	15 (68)
Interleukin-2	4 (18)
Biochemotherapy	3 (14)
Other	3 (14)

Some patients received more than one treatment.

**Table 5:** Patients with cutaneous melanoma after first ipilimumab.

Patient	OS (months)	Status	Following therapies
2	11, 6	Dead	None
4	35, 1	Dead	None
8	85, 8	Dead	None
14	57, 4+	No evidence of disease	None
16	88, 3+	No evidence of disease	Anti-PD-1
18	77, 3+	Alive with disease	Anti-PD-1; MEK inhibitor combined with BRAF-inhibitor^*∗*^
19	15, 3	Dead	None
20	133, 5+	No evidence of disease	None
21	52, 7	Lost to follow-up	Unknown
22	80, 5+	No evidence of disease	None

^*∗*^MEK inhibitor was discontinued due to retinal vein occlusion.

## Data Availability

Data are available at https://doi.org/10.1155/2021/5531864.
